# Thermodynamic and Kinetic Binding Behaviors of Human Serum Albumin to Silver Nanoparticles

**DOI:** 10.3390/ma15144957

**Published:** 2022-07-16

**Authors:** Jinjun Tian, Zhenghai Shi, Gongke Wang

**Affiliations:** 1 Biological and Chemical Engineering, Nanyang Institute of Technology, Nanyang 473004, China; 3171007@nyist.edu.cn; 2 School of Materials Science and Engineering, Henan Normal University, Xinxiang 453007, China; 3 Key Laboratory of Green Chemical Media and Reactions, Ministry of Education, Collaborative Innovation Center of Henan Province for Green Manufacturing of Fine Chemicals, School of Chemistry and Chemical Engineering, Henan Normal University, Xinxiang 453007, China

**Keywords:** silver nanoparticles, human serum albumin, adsorption, thermodynamics, kinetics

## Abstract

A nanoparticle, under biological milieu, is inclined to be combined with various biomolecules, particularly protein, generating an interfacial corona which provides a new biological identity. Herein, the binding interaction between silver nanoparticles (AgNPs) and human serum albumin (HSA) was studied with transmission electron microscopy (TEM), circular dichroism (CD), and multiple spectroscopic techniques. Due to the ground state complex formed mainly through hydrophobic interactions, the fluorescence titration method proved that intrinsic fluorescence for HSA was probably statically quenched by AgNPs. The complete thermodynamic parameters were derived, indicating that the interaction between HSA and AgNPs is an entropy-driven process. Additionally, synchronous fluorescence and CD spectrum results suggested the conformational variation it has upon binding to AgNPs and the α-helix content has HSA visibly decreased. The kinetic experiments proved the double hysteresis effect has in HSA’s binding to the AgNPs surface. Moreover, the binding has between HSA and AgNPs follows the pseudo-second-order kinetic characteristic and fits the Freundlich model for multilayer adsorption. These results facilitate the comprehension about NPs’ underlying biological effects under a physiological environment and promote the secure applications of NPs biologically and medically.

## 1. Introduction

Nanotechnology, a major technological breakthrough in human understanding that is changing the world of nanotechnology, also brought great opportunities for the development of nanobiomedicine. However, there is a remarkable gap and limited understanding of the physico-chemical properties of NPs in the physiological system [1]. Physiological conditions influence the interaction of biological systems with NPs, which can describe the fate and biosafety of NPs. This information particularly reveals the sustained circulation phenomenon of NPs or the possible clearance mechanism by the immune system. Thus, clinical translation and success of NPs will depend on key interactions with proteins, DNA, organs, and tissues, etc., in the human body.

In particular, due to the sterilization ability, [1,2] the research and application of AgNPs in biological systems has received widespread attention [3,4]. As we all know, NPs are enshrouded by a layer of biomolecules after being exposed to a biological matrix, generating a novel interface called ‘protein corona’, thus changing the physicochemical properties of NPs to influence their biological effect [5,6]. In general, the competitive binding between NPs and proteins is a dynamic process. Its composition and surface properties determine the degree and specificity of their binding to proteins. Therefore, investigating the relationship between NPs and proteins will help us develop safe and efficient biomaterials.

NPs have significant effects on biological systems (such as proteins and DNA) [7,8]. Exploring the interaction of NPs with proteins and the subsequent biological effects is beneficial for understanding the physiological response of NPs in biological systems. Lai et al. systematically studied the protein corona formed on 20 nm silver and gold NPs through different surface modifications in human plasma [9]. The results revealed the unique binding pattern between silver and gold NPs and the plasma protein, an important role of the surface charge and metal core in determining protein corona composition. Mukherjee’s research group established an effective approach to lowering aggregation of NPs through controlling the concentration of protein and pH value of the solution. It was demonstrated that biofunctionalized AgNPs featuring decreased aggregation are more appropriate for bio-sensing applications [10]. Kennedy found that the incubation of 20 nm AgNPs using human serum albumin (HSA) stabilized the aggregation and dissolution of NPs. Further studies proved the role of NPs in reducing toxicity and cellular uptake in comparison with other 20 nm particles [11]. Ban and Paul reported that protein coated AgNPs could vary in the bovine α-lactalbumin structure as well as drastically decrease its bactericidal ability. However, in case that AgNPs were functionalized by a proper group represented by polyethylene glycol (PEG), the conformational changes in protein were prevented in retaining their antibacterial capacity [12].

Human serum albumin (HSA) is the richest protein in plasma that exerts significant impacts on numerous endogenous and exogenous ligands in transportation, distribution, and metabolism [13,14]. The functions of HSA depend highly upon its structure. Since albumin proteins may lose their original structure and function after adsorption on NPs surfaces, it is important to further understand how the structural integrity in albumin contributes to the efficient use of albumin as a drug carrier on NPs. Unluckily, there still lacks a good understanding about the factors influencing the interaction of HSA with NPs, particularly how surface ligands on NPs and intermolecular interactions of HSA molecules in corona formation influence the dynamics and conformation [15]. With the specific structural characteristics and physiological functions of HSA, it is usually employed as a model protein in research regarding the interaction with drug molecules, nanoparticles, and other ligands [16,17,18]. Studies have shown that the overwhelming majority of nanoparticles can change the conformation of protein, resulting in the disorder of protein function. This is the so-called “nano toxicity” [19,20,21]. The small particle size of NP makes it have a strong penetrating power, which is harmful for the organisms and environment. Consequently, this will be an opportunity and a challenge for the development of nanotechnology in environmental health and safety areas [22,23].

In the present work, to explain the action forces and adsorption behavior in HSA binding to the AgNPs surface, the molecular interaction between HSA and AgNPs was carried out by adopting multiple spectroscopy approaches. Through the systematic study, we aim at elucidating the specific binding mechanism and conformational change of HSA upon binding interaction with AgNPs [24]. Hence, this study has significant implications by offering meritorious information concerning the essence of interaction between protein and NPs and the physiological response of NPs in biological systems.

## 2. Materials and Methods

### 2.1. Materials

This study used silver nitrate from TianDa Chemical Reagent Co., Ltd. (Tianjin, China), whereas trisodium citrate from Alfa-Aesar (Ward Hill, MA, USA). Meanwhile, we acquired 99% HSA in Solarbio (Beijing, China), which could be applied as received. We also fabricated HSA stock solution (1.0 × 10^−4^ M) through dissolution of HSA within 0.01 M PBS (10 mL, pH 7.4, Suzhou kechuang biological technology Co., Ltd., Suzhou, Jiangsu, China). Besides, we employed a pHS-2C pH-meter (Shanghai Dapu Instruments Co., Ltd., Shanghai, China) to measure under ambient temperature. We applied doubly distilled water during this process, and preserved stock solutions under 0~4 °C in the dark.

### 2.2. AgNPs Synthesis and Feature Analysis

We fabricated AgNPs using Munro’s approach [25]. Generally, 0.018 g solid silver nitrate was introduced into 100 mL double-distilled water under 45 °C. After being boiled in a water solution, we instilled trisodium citrate (2 mL, 1%). Thereafter, we shook the boiling solution in 30 min again, then preserved it to be the stock solution. To prevent AgNPs oxidation and aggregation, we encapsulated stock solution for AgNPs under 4 °C within the refrigerator, followed by preservation for 4 days. Some experimental processes could not be completed with 4 days, so we re-synthesized AgNPs with the same method to keep the reliability of our experiments. When preparing AgNPs, all the glassware was rinsed using freshly fabricated aqua regia (Shanghai Sinopharm Chemical Reagent co., Shanghai, China) and washed completely with double distilled water before usage. Size, morphology, and chemical compositions characterization for AgNPs was made via a TU-1810 spectrophotometer (Puxi Analytic Instrument Ltd., Beijing, China), powdery X ray diffraction (XRD, Bruker D8 diffractometer with Cu Ka radiation, Germany), and TEM (transmission electron microscope, JEM 2100, Tokyo, Japan). For XRD measurement, the centrifuged AgNPs colloid was dropped directly onto glass sheets and dried in a vacuum drying oven. The sample was then repeatedly added and dried, until a layer of solid AgNPs was formed on the glass sheet. Finally, the prepared sample was characterized with XRD.

### 2.3. Fluorescence and Synchronous Fluorescence Detection

This work recorded fluorescence measurements with a fluorescence spectrophotometer (Varian, CARY Eclipse). Later, we set the emission and excitation wavelengths at 290–550 and 280 nm, respectively, in the slit width of 5 nm/5 nm, with the mean from three scans being determined for every spectrum. This study acquired HSA’s fluorescence emission with and without AgNPs, at an AgNPs concentration of 0~1.0 × 10^−10^ M. To assess how the temperature affected the binding of HSA to AgNPs, three sets of fluorescence measurements were carried out under various temperatures (293, 298, 303, and 310 K). A SHPDC-0515 thermostatic bath was used for the temperature control.

In addition, synchronous fluorescence spectra of protein provided typical data for tryptophan and tyrosine residues, separately. With a wavelength difference (Δλ = λ_em_ − λ_ex_) of 60 or 15 nm, this work determined HSA’s synchronous fluorescence spectra for analyzing protein conformational change.

### 2.4. UV-Vis Absorption Spectroscopy Measurements

All UV-vis absorption measurements of AgNPs (1.0 × 10^−4^ M) with and without HSA were recorded from 350 to 500 nm under ambient temperature via a TU-1810 spectrophotometer (Puxi Analytic Instrument Ltd., China). In addition, the quartz cuvette was applied using the 1.0 cm path length.

### 2.5. Circular Dichroism (CD) Detection

In the constant nitrogen flow, the CD spectra were measured via a Chirascan spectropolarimeter (Applied Photophysics Ltd., Leatherhead, England). To be specific, this study applied the quartz cell (1.0 cm, path length) at 190~260 nm under ambient temperature. Then, we averaged values in three measurements as the final spectrum with the mode of continuous scans. The bandwidth, scan rate, and response time were 1.0 nm, 10 nm/min, and 2 s, respectively. HSA content reached 1 × 10^−6^ M, while the content of AgNPs reached 0, 2.0 × 10^−11^, 6.0 × 10^−11^, and 2.0 × 10^−10^ M, separately, within the 0.01 M PBS (pH 7.40). The final CD spectra were obtained by subtracting the buffer contribution from the original protein spectra, and all measurements were repeated in triplicate.

## 3. Results and Discussion

### 3.1. Characterization of AgNPs

**Figure 1:**
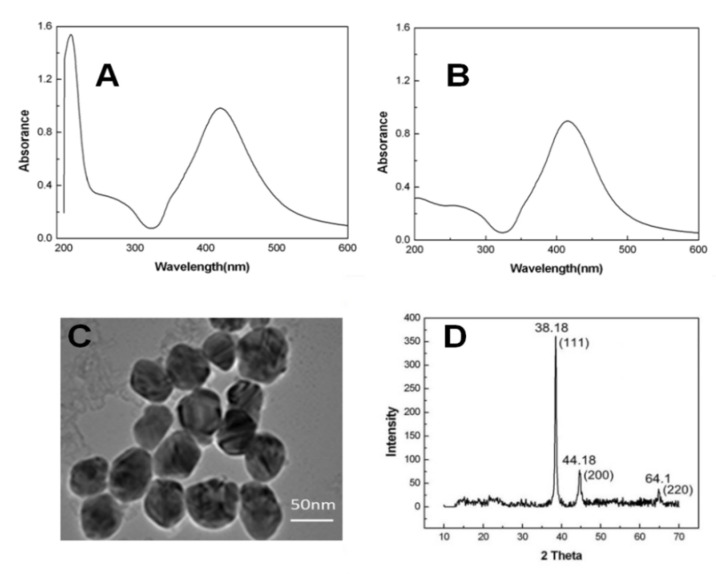
(**A**) UV-vis absorption spectra of freshly prepared AgNPs; (**B**) UV-vis absorption spectra of AgNPs after centrifugation; (**C**) Transmission electron microscope (TEM) image of prepared AgNPs; (**D**) XRD diagram of prepared AgNPs. The average dimensions of the AgNPs were decided by counting 60 NPs for each sample.

The UV-vis absorption spectrum was adopted to investigate the morphological characteristics of NPs. According to the surface plasmon resonance absorption peak location and the intensity in freshly prepared AgNPs, size and dispersity are explored (Figure 1A). The plasma absorption peak of AgNPs is located at 421 nm, and a similar result was reported in the previous work [26]. Figure 1B exhibits the absorption spectrum upon centrifugation. The maximum absorption shows an approximately 4 nm blue shift for AgNPs, while that for citrate (210 nm) does not exist. Based on the obtained results, citrate is possibly detached from the AgNPs surface, causing AgNPs aggregation partly. AgNPs were detected using TEM in size, morphology, and structure (Figure 1C). The prepared AgNPs are mostly spherical in shape and have good dispersion. Meanwhile, based on the TEM image, the average particle size of AgNPs is evaluated to be 40 ± 5 nm [27,28]. XRD is often used to investigate the microstructure of solid or some nonsolid substances. The XRD spectrum of the prepared AgNPs revealed three diffraction peaks at 64.1°, 44.1°, and 38.1° in association with planes {220}, {200}, and {111} in the face-centered cubic silver (Figure 1D). Based on the Scherr equation, the average particle size of AgNPs prepared as a solid sample was estimated at 35 nm, conforming to TEM micro-photograph findings [29].

### 3.2. Fluorescence Quenching Mechanism of HSA by AgNPs

Fluorescence quenching usually indicates the process which reduces the fluorescence intensity in the fluorophore. It can be induced by collisional diffusion-caused dynamic quenching between the fluorophore and quencher, or static quenching generating from ground state complex formation between the fluorophore and quencher [30]. Dynamic quenching appears in the case that excited state fluorophore is deactivated after contact with the quencher molecule from the solution. Under such circumstance, the fluorophore recovers the ground state in the diffusive encounter with the quencher. Static quenching appears after ground state complex formation between the fluorophore and quencher. According to the distinctive reliance on temperature and lifetime in the excited state, there exists differentiated dynamic and static quenching. In general, the higher temperature can induce faster diffusion and a higher chance of collision, which will accelerate the dissociation of the complexes. On this basis, the dynamic quenching constants increase with an increasing temperature. Comparatively, an increasing temperature may cause instability to complexes and may further decrease static quenching constants [31,32].

To further disclose the quenching mechanism of HSA with AgNPs, the Stern–Volmer equation was applied in quenching data analysis [33]:

(1) $$\frac{F_{0}}{F}=1+k_{q}\tau_{0}\left[Q\right]=1+K_{SV}\left[Q\right]$$

where *F*_0_ and *F* denote the steady-state fluorescence intensities in the protein without and with the quencher (AgNPs), [*Q*] means the concentration of the quencher, *k_q_* represents the bimolecular quenching rate constant, *K_SV_* signifies the Stern–Volmer dynamic quenching constant decided using linear regression in the Stern–Volmer equation and *τ*_0_ represents the average fluorophore life-time without the quencher.

**Figure 2:**
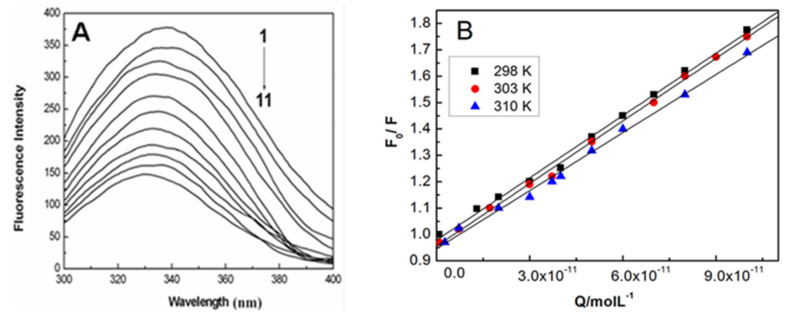
(**A**) The fluorescence spectra of HSA in the absence and presence of different concentrations of AgNPs; (**B**) Stern–Volmer plots for the HSA–AgNPs system at varying temperatures. The concentration of HSA was 1.0 × 10^−6^ M with the concentrations of AgNPs being (1–11): 0–1.0 × 10^−10^ M.

The HSA concentration was set to 1.0 μM, and the AgNPs concentration fluctuated at 0~1 × 10^−10^ M. The fluorescence emission spectra for HSA with and without AgNPs after excitation at 298 K are presented in Figure 2A. With an increasing AgNPs concentration, the emission intensity in HSA reduces progressively, accompanied by the blue shift of around 5 nm, which indicates the efficiency of AgNPs in quenching the intrinsic fluorescence for HSA. In addition, the palpable blue shift in fluorescence emission is possibly due to the conformational change in HSA. Figure 2B illustrates the diagrams for *F*_0_/*F* versus [*Q*]. Table 1 demonstrates the Stern–Volmer quenching constants *K_SV_* and quenching rate constants *k_q_* under various temperature conditions. As shown in Table 1, quenching constants *K_SV_* decreases with the increase in temperature, demonstrating that fluorescence quenching may originate from ground state complex formation between AgNPs and HSA. Moreover, the quenching rate constants *k_q_* is obviously higher than 2.0 × 10^10^ M^−1^·s^−1^, the maximal diffusion constant for biomolecules. Based on the above findings, it is suggested that the interaction between HSA and AgNPs possibly belongs to static quenching [34,35].

### 3.3. Binding Mode of HSA with AgNPs

**Figure 3:**
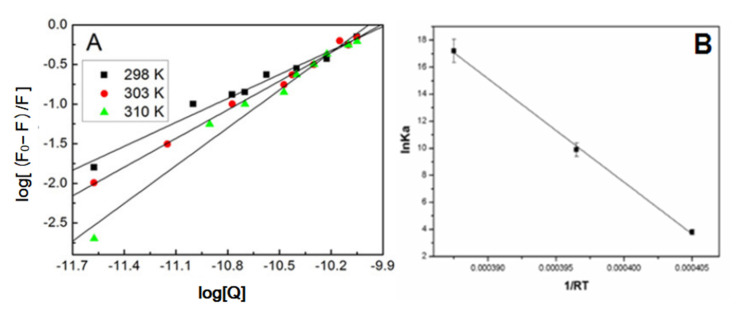
(**A**) Double logarithmic plots for the quenching of HSA by AgNPs at different temperatures; (**B**) Van’t Hoff plot for the interaction of AgNPs with HSA.

In static quenching, to probe the binding pattern between AgNPs and HSA, the fluorescence quenching process was explored with the equation below [36]:

(2) $$\log\frac{F_{0}-F}{F}=\log K_{b}+n\log[Q]$$

where [*Q*] indicates the quencher concentration, *n* refers to the number of binding sites, and *K_b_* denotes the binding constant. With the linear regression of a plot of log ((*F*_0_ − *F*)/*F*) versus log [*Q*] (Figure 3A), the binding parameters of the interaction of AgNPs with HSA under diverse temperature conditions can be found and presented in Table 2. The values of *K_b_* can reach in the order of 10^4^ M, and increase gradually with the increasing temperature, which demonstrates the conducive role of higher temperature in the binding of HSA to the AgNPs surface. In this respect, it is implied that the specific binding behavior of AgNPs to HSA may be a multiplayer adsorption process.

The acting forces between the ligands and the biomacromolecules mainly included hydrogen bonds, van der Waals forces, electrostatic, as well as hydrophobic interaction. To understand the energetic variations int he binding of HSA to AgNPs, the thermodynamic parameters in the binding process were assessed with the Van’t Hoff equation [37]:

(3) $$\ln K_{a}=-\frac{\Delta H^{0}}{RT}+\frac{\Delta S^{0}}{R}$$

The thermodynamic parameters (Δ*H*^0^, Δ*S*^0^) are determined with the Van’t Hoff equation, if the enthalpy (Δ*H*^0^) has little change over temperatures, then *K_a_* means the binding constant of the consistent temperature, *T* suggests the experimental temperature and *R* indicates the universal gas constant. Figure 3B illustrates Van’t Hoff diagrams of the binding of HSA to AgNPs under various temperature conditions. Besides, the free energy change (Δ*G*^0^) is expressed by:

(4) $$\Delta G^{0}=\Delta H^{0}-T\Delta S^{0}$$

Based on Equations (3) and (4), the thermodynamic parameters (Δ*H*^0^, Δ*S*^0^, and Δ*G*^0^) for the binding of HSA to AgNPs under various temperature conditions were evaluated. Table 2 presents the final results. The negative value for Δ*G*^0^ progressively increases as temperature increases, which demonstrates that HSA adsorption on the AgNPs surface was favorable with the higher temperature. The positive value for Δ*S*^0^ indicates that the binding of HSA to AgNPs may due to hydrophobic and electrostatic forces. The enthalpy change (Δ*H*^0^) reflects the intermolecular bond energy, while the entropy change (ΔS*^0^*) usually reveals the disorder change during binding. According to Table 2, $\Delta H^{0}$ and $\Delta S^{0}$ are positive values, suggesting that the mainstream acting force in the interaction of AgNPs with HSA may be hydrophobic interaction [38,39,40]. Importantly, the results further suggest the entropy-driven property of the binding of AgNPs to HSA, and verify that binding affinities and temperatures are positively correlated in the binding process [41,42].

### 3.4. Conformational Changes of HSA

To study how nanoparticles affect the protein microstructure, fluorescence spectroscopy on synchronous mode and CD spectra were employed for revealing HSA’s conformational changes with AgNPs.

The synchronous fluorescence spectrum for HSA after AgNPs addition were illustrated in Figure S1. In this case, when the scanning interval (Δλ = λ_em_ − λ_ex_) is set 15 or 60 nm, the synchronous fluorescence spectroscopy for HSA will offer the characteristic information for the amino acid residues tyrosine (Try) or tryptophan (Trp), respectively [43,44,45]. The fluorescence emission peak in amino acid residues is greatly influenced by the microenvironment, so the shift in maximum emission wavelength shows the changes in polarity of the microenvironment surrounding Tyr or Trp residues [46,47]. With Δλ = 60 nm, the maximum emission peak in HSA witnessed a distinct blue shift (Figure S1B), which shows that the addition of AgNPs altered the microenvironmental polarity of Trp. Moreover, the polarity and hydrophilicity in Trp residues reduced, suggesting that Trp residues could hardly have exposure to aqueous solution. On the other hand, nearly no shift occurred at the maximum emission wavelength in HSA with Δλ = 15 nm (Figure S1A), implying the insignificant impact of AgNPs addition on the Tyr microenvironment. Therefore, the present results seem to demonstrate that the binding sites between HSA and AgNPs primarily existed in Trp residues.

**Figure 4:**
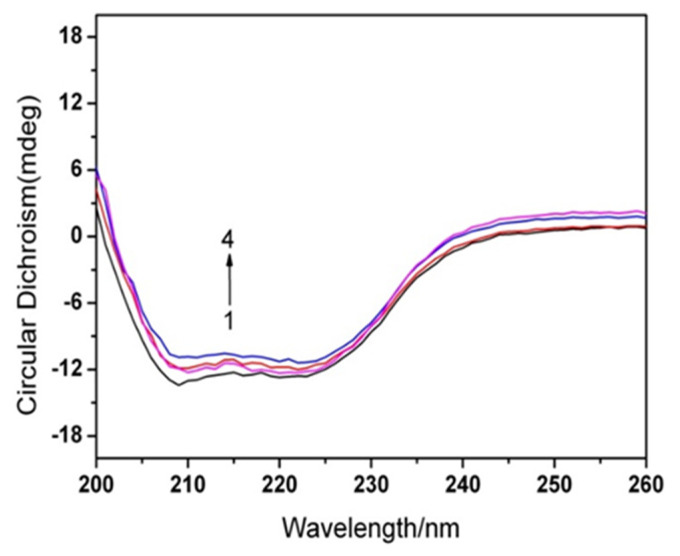
CD spectra of HSA in the absence and presence of AgNPs. The concentration of HSA is 1 × 10^−6^ M, the concentrations of AgNPs reach (1–4): 0, 1.5 × 10^−11^ M, 4.0 × 10^−11^ M, and 1.0 × 10^−10^ M, separately.

CD refers to a sensitive technology in monitoring the conformational changes in protein. As a result, the binding of AgNPs to HSA was also studied with CD spectra. Figure 4 shows the CD spectra for HSA without and with AgNPs. CD spectra for HSA had two negative bands at 208 and 222 nm, featuring the α-helix structure in the protein. Besides, the binding of AgNPs to HSA decreases negative ellipticity of the far UV CD spectra with no visible shift of absorption bands, indicating decreased α-helix content in HAS [48]. In this case, the percentage of α-helix content in HSA is measured as below [49]:

(5) $$\alpha -Helix(\%)=\left[\frac{-{\mathrm{MRE}}_{208}-4000}{33000-4000}\right]\times 100$$

where MRE_208_ refers to the measured mean residue ellipticity (MRE) value at 208 nm, and its value can be derived from Equation (6). Further, 4000 represents the MRE value for β-form and random coil conformation cross at 208 nm, and 33,000 represents the MRE value for the absolute α-helix conformation at 208 nm.

(6) $${\mathrm{MRE}}_{208}=\frac{\mathrm{observedCD}(\mathrm{mdeg})}{C_{p}nl\times 10}$$

where *C_p_* means the molar concentration in protein (HSA), n stands for the number of amino acid residues (583 for HSA) and *l* denotes the path length (1.0 cm). Besides, the α-helix content in the protein can be calculated with Equations (5) and (6). In the research, the α-helix content in native HSA was set at 55.48%, while α-helix content in HSA reduced to 54.21%, 53.65%, and 52.06% upon AgNPs addition at a concentration of 1.5 × 10^−11^ M, 4.0 × 10^−11^ M, and 1.0 × 10^−10^ M, separately. Consequently, these findings seem to confirm the results of synchronous fluorescence spectrum. The secondary structure contents in the protein are in a close relationship with its biological activities, indicating the loss of biological activity in HSA. In light of this, these observations suggest the conformational change of the protein at the secondary structural level in the binding between HSA and AgNPs [50].

### 3.5. Kineticstudieson the Adsorption Process

**Figure 5:**
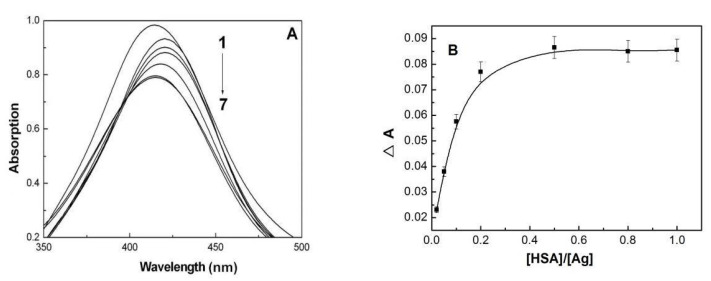
(**A**) The localized surface plasma resonance absorption of different concentrations of HSA with AgNPs, C(AgNPs) = 1.0 × 10^−4^ M, 1–7: [HSA]/[AgNPs] = 0, 1:50, 1:40, 1:30, 1:20, 1:10, 1:1; (**B**) The relationship between the change of absorption intensity (△A) and [HSA]/[AgNPs].

The interaction between AgNPs and HSA is supposed to be a dynamic adsorption process, the hysteresis impact is evaluated through the change of absorption peak intensity in AgNPs upon addition of the different concentrations of HSA. From Figure 5A, the absorption of AgNPs decreases successively, and experiences a red shift and then blue shift with the decrease of absorption intensity upon HSA addition. It suggests the gradual enhancement of HSA’s coating process on the AgNPs surface as HSA concentration increases. Besides, the absorption intensity in AgNPs is inclined to a platform upon HSA addition (Figure 5B), suggesting the saturation absorption of HSA on the AgNPs surface. From these observations, we can conclude that HSA has a significant hysteresis effect on AgNPs aggregation. Furthermore, with the continuous HSA adsorption on the AgNPs surface, the conformational change in HSA molecules is also a slow process [31]. Thus, these results illustrate that HSA adsorption on the AgNPs surface possess a typically double hysteresis effect.

The adsorption kinetics of HSA molecules with AgNPs is significant to predict the adsorption rate of protein molecules on the NPs surface in aqueous solutions and provides valuable data to better understand the adsorption reaction mechanism [51]. The optimal adsorption isotherm was determined when initial HSA concentration was 250 mg/L, with the AgNPs concentration of 20 mg/L. The size of synthesized AgNPs was 35 nm, while the size of the HSA molecule is about 3 nm, which is quite small compared to the AgNPs particle. Therefore, when HSA interacts with AgNPs, it is predicted that HSA molecules tend to adsorb on the surface of AgNPs with the physical or chemical types. At the different time intervals, the suspension was centrifuged with the HSA concentration of the supernatant being measured to study the adsorption kinetics in HSA molecules and AgNPs. The adsorption (*q_t_*) amount at time t was measured as [52,53,54].

(7) $$q_{t}=\frac{(C_{0}-C_{t})V}{W}$$

where *C*_0_ and *C_t_* (mg/L) stand for initial HSA concentration and HSA concentration at time *t*, *V* means the volume in the mixed solution (L), and *W* suggests the mass (mg) for the addition of AgNPs.

The kinetic adsorption process in HSA molecules on AgNP surfaces is often assessed with two fundamental kinetic models (pseudo-first-order or pseudo-second-order). In addition, the linear form for the pseudo-first-order kinetic equation can be indicated by:

(8) $$\log(q_{e}-q_{t})=\log q_{e}-\frac{k_{1}}{2.303}t.$$

where *q_e_* and *q_t_* refer to the absorption amount of HSA on AgNPs at equilibrium and at time *t*, separately. *k*_1_ means the equilibrium rate constant for pseudo-first-order adsorption.

**Figure 6:**
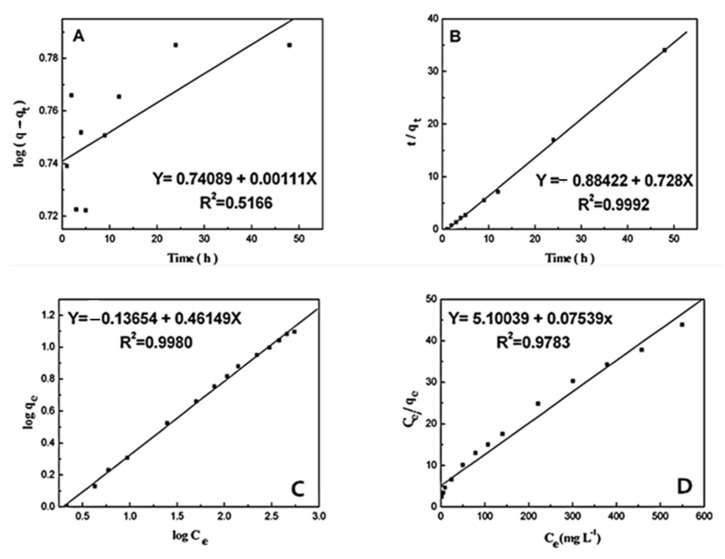
(**A**) The pseudo-first-order kinetics presented as a function of log(*q_e_ − q_t_*) versus time (*t*); (**B**) the pseudo-second-order kinetics presented as a function of *t/q_t_* versus time (*t*); (**C**) Freundlich isothermal adsorption of HSA on AgNPs surface; (**D**) Langmuir isothermal adsorption of HSA on the AgNPs surface.

In addition, the slope and intercept in the plot for log(*q_e_* − *q_t_*) versus *t* were employed for determining the pseudo-first-order rate constant *k*_1_ and equilibrium adsorption density *q_e_* (Figure 6A). The correlation coefficient (*R*^2^) values are employed for evaluating the dependence of linear relationship. It is shown that the correlation coefficient value (*R*^2^ = 0.5166) herein is comparatively small. Besides, the pseudo-first-order rate constant *k*_1_ and *q_e_* decided using the model suggest the inconsistency between the experimental *q_e_* value and measured value. Thus, the kinetic behavior for the binding of HSA to AgNPs possibly does not agree with the pseudo-first-order kinetic model.

The linear form in pseudo-second-order kinetic equation is presented as below:

(9) $$\frac{t}{q_{t}}=\frac{1}{k_{2}{\left(q_{e}\right)}^{2}}+\frac{t}{q_{e}}.$$

where *k*_2_ means the equilibrium rate constant for pseudo-second-order adsorption. According to slope and intercept, the second order rate constant *k*_2_ and *q_e_* values were decided (Figure 6B). According to Figure 6B, the correlation coefficient (*R*^2^ = 0.9992) indicates the strength in the linear relationship. Additionally, it seems that the theoretical *q_e_* value is perfectly consistent with that of the experimental value. As a result, it can be confirmed that HSA adsorption on AgNPs is in line with the pseudo-second-order kinetics.

The adsorption process of protein on NPs surfaces in biological fluid is usually portrayed using Freundlich and Langmuir isotherms. The Freundlich isotherm is an empirical formula, which is used on the assumption that protein molecules have multilayer adsorption on the surface of NPs. However, Langmuir isotherm is proposed according to the assumption that protein molecules have monolayer adsorption on NPs surfaces. The adsorption parameters of the HSA-AgNPs system were fitted with Freundlich and Langmuir adsorption equations linearly in order to investigate the optimal isothermal adsorption models. Figure 6C,D shows the adsorption isotherms for HSA on the AgNPs surface at room temperature. In this case, HSA and AgNPs represent the adsorbate and adsorbent, separately. Multilayer adsorption on NPs surfaces is measured using the Freundlich adsorption isotherm model, which can be given as the following equation:

(10) $$q_{e}=K_{F}C_{e}^{1/n}.$$

where *C_e_* represents the mass HSA concentration in the supernatant (mg/L), *q_e_* means the amount of HSA (mg) adsorbed per mg of AgNPs in the equilibrium, *K_F_* signifies the Freundlich constant (L^1/*n*^·mg^−1/*n*^), and 1/*n* denotes the heterogeneity factor. The linear form for the Freundlich expression is derived from Equation (10):

(11) $$\log q_{e}=\frac{1}{n}\log C_{e}+\log K_{F}.$$

The plot for log *q_e_* versus log *C_e_* is adopted for determining the constant *K_F_* and exponent 1/*n* (Figure 6C). The Freundlich isotherm suggests multilayer adsorption, which is not limited to the formation of monolayer. Therefore, the Freundlich equation estimates that the HSA amount on the AgNPs surface increases with the increasing concentration of HSA in the existing environment. Besides, the Freundlich isotherm constants were predetermined in our case. It is shown that the experimental data are a good fit for the Freundlich model, indicating the multilayer adsorption of HSA on the AgNPs surface. In addition, the high linear regression coefficient value (*R*^2^ = 0.9980) suggests the Freundlich model’s good predictability for HSA’s adsorption on AgNPs. As a result, the Freundlich constant and the heterogeneity factor are respectively *K_F_* = 1.051 L^1/*n*^ mg^−1/*n*^ and 1/*n* = 0.395.

The Langmuir isothermal adsorption model hypothesizes that in the case that a protein molecule takes up a site on the AgNPs surface, the site cannot continually adsorb more protein molecules. The Langmuir isotherm equation is denoted as below:

(12) $$q_{e}=\frac{q_{m}K_{a}C_{e}}{1+K_{a}C_{e}}.$$

where *C_e_* means the mass HSA concentration of the supernatant (mg/L), *q_e_* indicates the amount of HSA (mg) adsorbed per mg of AgNPs, *q_m_* represents the maximum adsorption amount of HSA on the AgNPs surface at the monolayer, and *K_a_* denotes the adsorption constant (L/mg), indicating HSA’s affinity on the AgNPs surface. Equation (12) may be rewritten in the linear form as below:

(13) $$\frac{C_{e}}{q_{e}}=\frac{C_{e}}{q_{m}}+\frac{1}{K_{a}q_{m}}.$$

Obviously, the *q_m_* and *K_a_* values were estimated with the linear plots for *C_e_*/*q_e_* versus *C_e_* (Figure 6D). To solve the simultaneous equations, the Langmuir isotherm constants were simultaneously measured. Apparently, the great differences between graphical solution and analytical solution prove the inconsistency between experimental data and the Langmuir model. The relatively lower linear regression coefficient value (*R*^2^ = 0.9783) suggests that the Langmuir isothermal adsorption model is not a good coincidence with HSA adsorption to AgNPs. Consequently, these results indicated that HSA adsorption on the AgNPs surface possibly conforms to the Freundlich isothermal adsorption model, and the binding of HSA to AgNPs is a multilayer adsorption process [55,56,57].

## 4. Conclusions

To conclude, the interaction between silver nanoparticles and human serum albumin was assessed using transmission electron microscopy. The binding of AgNPs to HSA is a multilayer adsorption process, and the adsorption is a second-order reaction. With these findings, in the future, we can also focus on various factors such as size, charge, and surface composition of NPs, facilitating the evaluation of the effect of various factors on the binding properties for NPs–proteins systems. The secure application for NPs in biological systems deserves more attention, which should be further studied to gain deeper insights. With the analysis of the interaction of AgNPs with HAS in different temperatures, the results implied the interaction may become a static quenching process. In particular, the measured thermodynamic parameters (Δ*G*^0^, Δ*H*^0^, and Δ*S*^0^) indicate that the binding process is spontaneous with the major acting forces being hydrophobic interactions. It was demonstrated that silver nanoparticles can influence the microenvironment for tyrosine and tryptophan residues of HSA molecules, enhance their hydrophobicity, and change the tertiary structure of HSA molecules by synchronous fluorescence spectrometry. With the UV-vis absorption spectrum adsorption process of HSA on silver nanoparticles’ surface, we found the pseudo-second-order kinetic characteristics and apparent hysteresis impact of HAS adsorption on the AgNPs surface. The HSA adsorption on the surface was also observed.

## Figures and Tables

**Table 1:** Ster–Volmer quenching constants of AgNPs and HSA at varying temperatures.

*T* (K)	*K_SV_* (10^9^ mol·L^−1^)	*K_q_* (10^17^ L·mol^−1^·s^−1^)	*R* ^a^	*SD* ^b^
298	7.639	7.639	0.9917	0.03625
303	7.443	7.443	0.9927	0.03324
310	7.274	7.274	0.9892	0.04161

^a^*R* is the correlation coefficient; ^b^ *SD* is the standard deviation.

**Table 2:** The binding constants and thermodynamic parameters of HSA/AgNPs systems at varying temperatures.

*T* (K)	*K_b_* (10^4^ L·mol^−1^)	*R* ^a^	*SD* ^b^	∆*H*^0^ (kJ·mol^−1^)	∆*G*^0^ (kJ·mol^−1^)	∆*S*^0^ (J·mol^−1^)
298	6.609	0.9940	0.0531	124.68	−28.71	514.73
303	38.616	0.9970	0.0437		−31.28	
310	67.098	0.9976	0.0407		−34.89	

^a^*R* represents the correlation coefficient of the binding constant *K_b_*; ^b^ *SD* denotes the standard deviation of the curve obtained by fitting according to Equation (2).

## Data Availability

Data underlying the results presented in this paper are not publicly available at this time but may be obtained from the authors upon reasonable request.

## Supplementary Materials

The following are available online at https://www.mdpi.com/article/10.3390/ma15144957/s1, Figure S1: Synchronous fluorescence spectra of HSA in the absence and presence of AgNPs at pH 7.4. (A) Δλ = 15 nm, the concentration of HSA was 1.0 × 10^−6^ M, and the concentrations of AgNPs were (1–14): 0–1.6 × 10^−10^ M; (B) Δλ = 60 nm, the concentration of HSA was 1.0 × 10^−6^ M, and the concentrations of AgNPs were (1–11): 0–1.0 × 10^−10^ M.
