# Supplementary material for: Thermodynamic and Kinetic Binding Behaviors of Human Serum Albumin to Silver Nanoparticles
Source: Materials (Basel). 2022 Jul 16;15(14):4957. doi: 10.3390/ma15144957 (PMC9323290; doi:10.3390/ma15144957)
Supplement: Supplementary file 1 [file materials-15-04957-s001.zip › materials-1740730-supplementary.pdf]

materials

MDPI

Supplementary Material

Thermodynamic and Kinetic Binding Behaviors of Human Serum Albumin to Silver Nanoparticles

Jinjun Tian [1], Zhenghai Shi [1,*] and Gongke Wang [2,3,*]

1 Biological and Chemical Engineering, Nanyang Institute of Technology, Nanyang 473004, China; 3171007@nyist.edu.cn
2 School of Materials Science and Engineering, Henan Normal University, Xinxiang 453007, China
3 School of Chemistry and Chemical Engineering, Key Laboratory of Green Chemical Media and Reactions, Ministry of Education, Collaborative Innovation Center of Henan Province for Green Manufacturing of Fine Chemicals, Henan Normal University, Xinxiang 453007, China
* Correspondence: 3131004@nyist.edu.cn (Z.S.); 031145@htu.cn (G.W.)

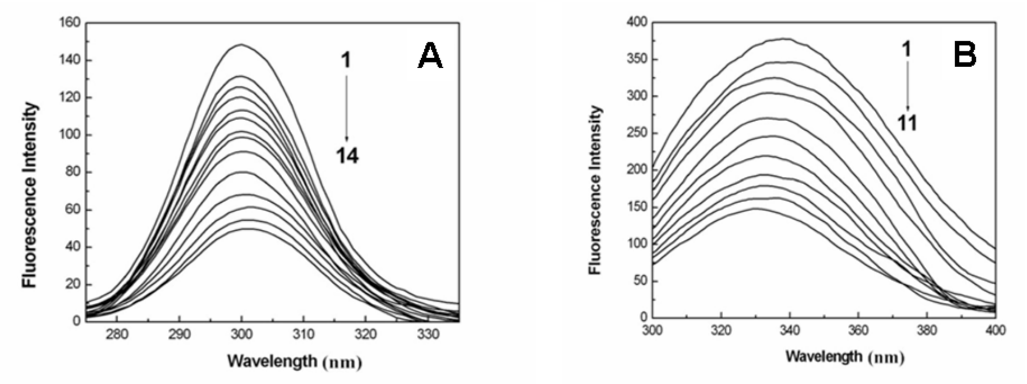

Figure S1. Synchronous fluorescence spectra of HSA in the absence and presence of AgNPs at pH 7.4. (**A**) $\Delta\lambda$ = 15 nm, the concentration of HSA was 1.0×10^{-6} M, and the concentrations of AgNPs were (1–14): 0–1.6×10^{-10} M; (**B**) $\Delta\lambda$ = 60 nm, the concentration of HSA was 1.0×10^{-6} M, and the concentrations of AgNPs were (1–11): 0–1.0×10^{-10} M.
